# Shortening the Time of the Identification and Antimicrobial Susceptibility Testing on Positive Blood Cultures with MALDI-TOF MS

**DOI:** 10.3390/diagnostics11081514

**Published:** 2021-08-23

**Authors:** Ya-Wen Tsai, Ting-Chia Lin, Hsiu-Yin Chou, Huei-Ya Hung, Che-Kim Tan, Li-Ching Wu, I-Jung Feng, Yow-Ling Shiue

**Affiliations:** 1 Department of Clinical Pathology, Chi-Mei Medical Center, Tainan 71004, Taiwan; rositatsai@gmail.com (Y.-W.T.); u104007417@cmu.edu.tw (T.-C.L.); 540014@mail.chimei.org.tw (H.-Y.C.); gill9091@hotmail.com (H.-Y.H.); 540012@mail.chimei.org.tw (L.-C.W.); 2 Institute of Biomedical Sciences, National Sun Yat-sen University, Kaohsiung 80424, Taiwan; 3 Institute of Precision Medicine, National Sun Yat-sen University, Kaohsiung 80424, Taiwan; 4 Department of Intensive Care Medicine, Chi-Mei Medical Center, Tainan 71004, Taiwan; chekim.tan@gmail.com

**Keywords:** bacterial identification, antimicrobial susceptibility tests, septic shock

## Abstract

The current processes used in clinical microbiology laboratories take ~24 h for incubation to identify the bacteria after the blood culture has been confirmed as positive and fa further ~24 h to report the results of antimicrobial susceptibility tests (ASTs). Patients with suspected bloodstream infection are treated with empiric broad-spectrum antibiotics but delayed targeted antimicrobial therapy. This study aimed to develop a method with a significantly shortened turnaround time for clinical application by identifying the optimal incubation period of a subculture. A total of 188 positive blood culture samples obtained from Nov. 2019 to Aug. 2020 were included. Compared to the conventional 24-h incubation for bacterial identification, our approach achieved 96.1% and 97.4% identification accuracy after shortening the incubation time to 4.5 and 3.5 h for gram-positive (GP) and gram-negative (GN) bacterial samples, respectively. Samples from short-term incubation without any intermediate step or process were directly subjected to analysis with the Phoenix M50 AST. Compared to the conventional disk diffusion AST, the category agreements for GP (excluding *Streptococcus* spp.), *Streptococcus* spp., and GN bacterial samples were 91.8%, 97.5%, and 92.7%, respectively. Our approach significantly reduced the average turnaround time from 48 h to 28 h for reporting bacterial identity and decreased average AST from 72 h to 50.3 h compared to the conventional methods. Accordingly, this approach allows a physician to prescribe the appropriate antibiotic(s) ~21.7 h earlier, thereby improving patient outcomes.

## 1. Introduction

Serious bloodstream infections trigger a dysregulated host inflammatory response to infection, leading to organ dysfunction or sepsis. Sepsis is a life-threatening syndrome with a mortality rate ranging from 20% to 50% that affects more than 30 million people worldwide every year, potentially causing six million deaths [1]. In addition to high mortality, sepsis survivors suffer from a myriad of physiological, physical and psychological challenges [2]. Studies have demonstrated that antibiotic treatments within 6 h upon infection significantly decreases in-hospital mortality rates [3], while the survival rate falls by approximately 7.6% with a 1 h delay for antimicrobial administration [4].

Administering antibiotic treatment through an empirical antibiotic treatment to patients with bloodstream infections shortens the length of hospital stay and lowers the fatality rate when compared to those who are prescribed with inappropriate therapies [5,6]. A blood culture is a test that identifies blood infection in a clinical process involving sample collection, incubation, identification (ID), and antimicrobial susceptibility testing (AST). The conventional ID method for blood culture requires at least an overnight incubation and a series of subsequent biochemal tests to identify the profile of the sampled microorganisms. Studies have shown that, from the time of specimen collection, it usually takes one day to determine Gram-positive (GP) and Gram-negative (GN), two days to identify the pathogen, and three days to obtain antimicrobial susceptibility test (AST) reports [7,8]. In other words, it takes at least three days for a suspected case to access the appropriate antibiotics. A delay in any step may result in negative consequences to the patient’s clinical outcomes. Due to the time-consuming and labor-intensive nature of conventional methods, new technologies and alternative procedures have been developed to shorten the ID and AST reporting times. Matrix-assisted laser desorption ionization-time of flight mass spectrometry (MALDI-TOF MS) emerges as one of the technologies most commonly utilized in the clinical setting for pathogen identification and numerous laboratory-developed protocols [9,10,11,12].

Antibiotic stewardship programs have promoted awareness of the usage of antibiotics and advanced diagnostic tools in the clinical laboratory; however, several medical needs in bloodstream infection management remain unmet. It is a common goal for global clinical researchers and scientists to shorten the AST reporting time. Thus, we proposed a direct AST method without subculture procedures that included the centrifugal separation [12,13,14], blood cell lysis and filtration [15], and stable-isotope labeling [16] to avoid extra manual or bioinformatics steps. Our study aimed to shorten incubation time for ID and AST, thereby optimizing routine procedures in clinical microbiology laboratories.

## 2. Materials and Methods

### 2.1. Sample Collection and Culture

Blood samples were collected from patients with suspected bacteremia, sepsis or systemic inflammatory response syndrome (SIRS) between Nov. 2019 and Aug. 2020 at the Chi-Mei Medical Center, Tainan, Taiwan. Each blood culture set contained one aerobic and one anaerobic bottle, respectively. Collected samples (8–10 mL) were incubated with the BD BACTEC™ FX blood culture system (BD, Franklin Lakes, NJ, USA) at 35 °C. Once the culture was identified to be positive, Gram staining and subculturing were undertaken.

### 2.2. Subculture and Identification of Bacterial Samples

Positive samples were subcultured on the BAP/EMB bi-plate (BBL™Trypticase™ Soy Agar with 5% Sheep Blood/Levine EMB Agar, BD) and further incubated overnight at 35 °C with CO_2_. The bacterial colonies were subsequently picked for ID by MALDI-TOF MS (Bruker Daltonik, Bremem, Germany) and followed by AST with a BD Phoenix™ M50. When comparing the peptide mass fingerprint of unknown samples to the Bruker Biotyper reference database (Bruker, Billerica, MA, USA), a similarity score > 1.7 [percentage of reliable ID (%)] indicates a match to the optimal genus-level, thereby rendering practical indication for clinical treatment.

### 2.3. The AST Using the BD Phenix^TM^ M50 Automated Microbiology System

An automated microbiology system, the BD Phoenix^TM^ M50, including different panels intended for in vitro rapid ID and AST, was used. The selected panels (NMIC-411, PMIC-95 and SMIC/ID-8) were used to determine minimum inhibitory concentration (MIC). The AST method used in the BD Phoenix M50 system is a broth-based microdilution test. It utilizes a redox indicator for the detection of organism growth in the presence of an antimicrobial agent. Continuous measurements of changes to the indicator as well as bacterial turbidity are used in the determination of bacterial growth. Every AST panel configuration contains several antimicrobial agents with a wide range of two-fold doubling dilution concentrations. Organism identification by MALDI-TOF MS is used for the interpretation of the minimum inhibitory concentration (MIC) values of each antimicrobial agent.

The AST broth was poured into a selected panel, which was next placed into the instrument to incubate for 16 h. The colonies from overnight incubation were tested using the BD Phoenix^TM^ M50 AST system with priority given to critically important antibiotics, including NMIC-411, PMIC-95, and SMIC/ID-8 panels, which contain 15 antimicrobials for GN bacteria, 11 antimicrobials for non-*Streptococcus* GP bacteria and 5 antimicrobials for *Streptococcus*, respectively. M50 AST not only provides AST (Sensitive: S/Resistant: R) results but also automatically detects MIC, reducing the reading bias of diameter measurements which were caused by different laboratory operators using the conventional disk diffusion AST system.

### 2.4. Short-Term Incubation Process

Positive blood culture samples were subcultured to a plate and incubated for 1.5, 2.0, 2.5, 3.0, 3.5, 4.5 and 24 h, respectively. At each time point, colonies were picked up for identification by MALDI-TOF MS. The GN and GP colonies which were incubated for 3.5 h and 4.5 h, respectively, were subject to an AST test with the BD Phoenix^TM^ M50 system.

### 2.5. Quality Control

Standard strains including *Staphylococcus aureus* ATCC 29213, Staphylococcus saprophyticus ATCC BAA-977, *Streptococcus pneumoniae* ATCC 49613 and *Enterococcus faecalis* ATCC 29212 were used for internal quality control among GP. Furthermore, standard *Escherichia coli* ATCC 25922 and *Pseudomonas aeruginosa* ATCC 27853 were used for internal quality control strains among GN. The ID results adhered to the criteria that were established for routine practice by the ISO 15189 accredited microbiology laboratory in Chi-Mei Medical Center. In addition, the analysis of short-term incubation samples and overnight incubation samples for AST were consistent and met the quality control requirements of Phoenix^TM^ panels.

### 2.6. Statistical Analysis

A chi-square test or Fisher’s exact test was used to identify the number of samples displaying a reliable ID from short-term incubation and overnight incubation (the conventional method) as appropriate. In the AST, the consistency of antimicrobial susceptibility (S) or resistance (R) status reported from colonies of short-term incubation on the M50 AST panels (the proposed method in this study). The conventional overnight colonies on disk diffusion AST were also investigated to assess their accuracy when compared to the proposed method. According to the CLSI guideline M52 (verification of commercial microbial identification and antimicrobial susceptibility testing systems, CLSI) [17], consistency is evaluated from the agreement between the proposed and the conventional methods. Results included the following categories: category agreement (CA, agreement between the proposed and the conventional method), very major error (VME, false susceptibility), major error (ME, false resistance), and minor error (susceptible/resistant vs. intermediate susceptibility). Furthermore, the impact of the shortened incubation time on report agreement between the M50 AST panels and the conventional disk diffusion AST was examined by calculating of the CA. A *p* value < 0.05 is considered as statistically significant.

## 3. Results

### 3.1. The Optimal Incubation Time for ID

**Figure 1:**
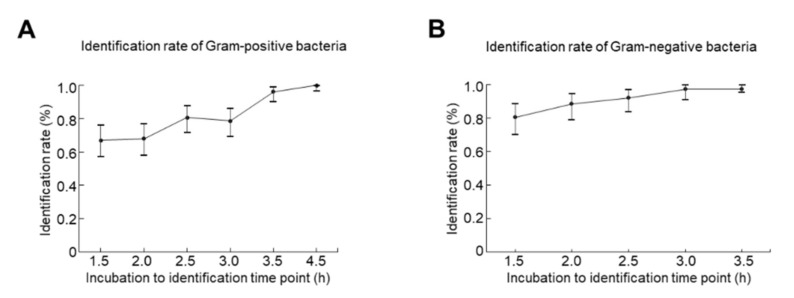
Identification rates (point estimations and 95% confidence intervals) for (**A**) Gram-positive and (**B**) Gram-negative bacteria at different incubation time periods.

A total of 188 blood cultures with positive responses (180 monomicrobial and 8 polymicrobial) were included in this study, and 103 GP and 77 GN bacteria were subsequently isolated. The accuracy rate for ID of GP bacteria after 1.5-, 2-, 2.5-, 3-, 3.5-, and 4.5-h incubation were identified as 67.0% (69/103, *p* < 0.0001) (95% confidence interval [CI]: 57.0 to 75.9%), 68.0% (70/103, *p* < 0.0001; 95% CI: 58.0 to 76.8%), 80.6% (83/103, *p* < 0.0001; 95% CI: 71.6 to 87.7%), 78.6% (81/103, *p* < 0.0001; 95% CI: 69.5 to 86.1%), 96.1% (99/103, *p =* 0.1214; 95% CI: 90.4 to 98.9%), and 100% (103/103, *p >* 0.9999; 95% CI: 96.5 to 100%) compared to those from the conventional method (Table 1). Due to the delayed growth of *Streptococcus* spp., the detection was not performed at the specific time point shown as ‘-’. Those organisms were not able to be identified (similarity score < 1.7) shown as ‘0′. Accordingly, the optimal incubation time to ascertain GP bacterial ID was 4.5 h (Figure 1A). On the other hand, the identification rates for GN bacteria after 1.5-, 2-, 2.5-, 3-, and 3.5-h incubation were 80.5% (62/77, *p* = 0.0003; 95% CI: 69.9 to 88.7%), 88.3% (68/77, *p* = 0.0030; 95% CI: 79.0 to 94.5%), 92.2% (71/77, *p* = 0.0282; 95% CI, 83.8 to 97.1%), 97.4% (75/77, *p* = 0.0500; 95% CI: 90.9 to 99.7%), and 97.4% (75/77, *p* = 0.0500; 95% CI, 90.9 to 99.7%), respectively when compared to the conventional method (Table 2). The optimal incubation time to confirm GN bacterial ID was identified as 3.5 h (Figure 1B). Of note, *Enterococcus* spp. was 100% (29/29) identified after 2.5 h culture, which is the shortest time of incubation-to-identification among GP bacteria. Nevertheless, only 68.2% (30/44) of *Staphylococcus* spp. was detected after 2.5 h incubation. For GN bacteria, *Acinetobacter* spp., *Aeromonas* spp., *Leclercia* spp., *Morganella* spp., *Proteus* spp., and *Stenotrophomonas* spp. were all 100% identified after 1.5 h incubation, suggesting that shortening the incubation-to-identification time for GN bacteria is highly feasible.

### 3.2. The Short-Term Incubation on the M50 AST Panels Showed High Category Agreements Compared to the Conventional Disk Method

A total of 103 isolates of GP bacteria including 30 isolates of *Streptococcus* spp. (Table 1) and 77 GN isolates (Table 2) were assessed with 11, 5 and 15 antimicrobial agents, respectively (Table 3, Table 4 and Table 5). Compared to the conventional disc diffusion AST, the CA of the BD Phoenix M50 AST system by short-term incubation for GP (excluding *Streptococcus* spp.) was 91.8% (427/465), the VME and ME rates of GP bacteria were 7.5% (11/146) and 6.1% (19/314), respectively (Table 3). However, coagulase-negative *Staphylococcus* (CoNS), involving mostly skin contamination, accounted for all the VME rate among the GP isolates and 36.8% (7/19) of the ME rate (Table 3). The VME and ME were 0% and 3.2% (7/218) after excluding the CoNS isolates (Table 3). As shown in Table 4, of *Streptococcus* spp., the CA was identified as 97.5%. In addition, of GN isolates, the CA was identified as 92.7% (Table 5). 

### 3.3. Short-Term Incubation on the Disk Diffusion AST and the BD Phoenix^TM^ M50 AST Panels Showed High Category Agreements Compared to Overnight Incubation Colonies

Compared to overnight incubation on disk diffusion AST, the CA of short-term incubation on disk diffusion AST for GP bacteria (excluding *Streptococcus* spp.) was 95.9% (328/342). Of GP bacteria, the VME, ME and the minor error rates were detected as 2.9% (3/104), 3.4% (8/236) and 0.9% (3/342). The CA of *Streptococcus* spp., the VME, ME, and the minor rate was 97.5% (116/119), 0% (0/8), 1.8% (2/111) and 0.8% (1/119), respectively. Among GN isolates, the CA was determined as 98.4% (Table 6). Likewise, compared to overnight incubation, the CA of short-term incubation for GP (excluding *Streptococcus* *spp.*) was 95.6% (1357/1420) on the BD Phoenix M50 AST system, while VME, ME and minor error rate was 1.5% (8/551), 2.1% (17/820) and 2.7% (38/1420), respectively. The CA of *Streptococcus* spp., VME, ME and minor error rates were 94.9% (370/390), 5.0% (2/40), 2.9% (10/345) and 2.1% (8/390), respectively. In addition, of GN isolates, the CA, VME, ME and the minor rates were 96.8% (1245/1286), 0.8% (3/355), 1.5% (13/876) and 1.9% (25/1286), respectively (Table 6).

## 4. Discussion

In this study, we used the short-term incubations (3.5 h for GN and 4.5 h for GP) without any intermediate step or process in conjunction with a rapid MALDI-TOF MS method to ID the infected microorganisms. The CA are concordant with the conventional overnight culture. Lately, numerous technologies and protocols have been developed to accelerate the turnaround time for blood culture ID and AST. One of these is a molecular-based method that is combined with the multiplex pathogen-specific PCR. Indeed, multiplex PCR provides a platform for simultaneously detecting common causative bacteria and antimicrobial resistance genes in 1–1.5 h [18]. The BioFire®FilmArray® Blood Culture Identification Panel (a multiplexed PCR array) is an FDA-cleared commercialized product which is able to examine 43 targets associated with bloodstream infections and the results can be reported within 1 h from a positive blood culture. However, the limitation of testing targets, missing or late (same as the conventional method) reports for AST [19], high costs, and heavy labor are the major disadvantages. Recently, Felsenstein et al. (2016) have proposed a shorter length of stay (median: 1.5 d) and a reduction in hospital costs (median: US$3757) for patients admitted to the general pediatric units after implementing a rapid molecular assay (BC-GP) [20]. Nevertheless, the BC-GP assay can only examine 12 common GP and three resistance determinants, resulting in restrictions to the sensitivity of bacteria ID and AST. Alternatively, MALDI-TOF MS offers a high-throughput, sensitive and specific analysis for many applications in microbiology, including clinical diagnostics [21]. Verroken et al. applied an automated system to inoculate onto Columbia blood agars and to process after a 5-h incubation on a MALDI-TOF MicroFlex platform (BD). In a total of 925 positive blood culture bottles, 727 (81.1%) monomicrobial bacteremia episodes were found to be concordant with the validated identification techniques [22]. Therefore, we adapted and improved this method in our study.

More importantly, we also showed that no extra extraction steps were required in our protocols. Different methods for inoculation and process have been developed to shorten the incubation time required for subculture ID. By using MALDI-TOF MS for ID from bacterial cultures incubated on the Columbia blood agars, the control species identification at 24 h was achieved in 100% of Gram-positive aerobic cocci (GPC) and 97.6% of Gram-negative aerobic rods (GNR), and with ethanol/formic acid protein extraction in positive blood cultures, it was reduced to 3.1 h [10]. However, the extraction time was not included. The identification rates for GP and GN were 64% and 76.2%, respectively. Moreover, by applying pelleted samples after centrifugation streaking into four quadrants on a 37 °C pre-warmed BAP agar, Bhatti et al. identified 94% of the GN bacteria (*n* = 47) after 4 h of incubation and 100% of the GP bacteria (*n* = 87) after 6 h of incubation [11]. Altun et al. presented a study of 515 positive blood samples using a rapid culture method and was able to identify 82.3% of isolates at 5.5 h using MALDI-TOF, including 73.4% of GP and 93.4% of GN bacteria [23]. Although the above procedures more or less improved the ID efficiency, additional sample preparations and tedious procedures/steps were introduced, making these protocols impractical. Previous studies have also shown that GN bacteria have a relatively shorter growth time and a higher identification rate than GP bacteria [10,11,23], which is consistent with our findings, since GN exhibited a higher identification rate than GP at the same time of culture.

The swift spread of bacterial resistance to antimicrobials and the changing resistance mechanisms reduce the lifespan of novel antimicrobials. Therefore, intensive studies persistently focus on the improvement of rapid AST. Romero-Gómez et al. combined the MALDI-TOF MS direct identification method with the VITEK-2^®^ (bioMérieux Inc., Durham, NC, USA) antimicrobial susceptibility test to evaluate its reliability. Flagged positive by the BACTEC™, blood culture samples were directly identified by MALDI-TOF, and followed with inoculation of VITEK-2^®^ AST cards, a turbidimetric method for automated susceptibility testing that is commercially available. The average time required to obtain the AST results for GN and GP was 6.45 ± 1.52 h and 9.55 ± 2.97 h respectively, which was shorter than our protocols. Positive cultures for GN bacteria were assessed with 19 antimicrobial agents and showed an agreement of 96.67%, with 2.09% of minor error, 0.72% of ME, and 0.50% of VME for the *Enterobacteriaceae* group (*n* = 231). Meanwhile, 67 isolates of GP were assessed for 19 antimicrobial agents, and agreement with all antimicrobial agents was 97.84%, with 1.24% of minor error, 0.49% of ME, and 0.41% of VME [13]. Barnini et al. proposed a novel method using Alfred 60AST (AlifaxSpA, Polverara, PD, Italy) to provide AST results in 6 h. Positive blood cultures were transferred to the serum separator and centrifuged to obtain a bacterial sediment, and suspensions of 0.9 McFarland in HB&L broth (Alifax) were subsequently produced and analyzed. Positive cultures for the *Enterobacteriaceae* were assessed with five antimicrobial agents and showed an agreement of 88.1%, with 3.3% of minor error, 17.6% ME, and 0% of VME (*n* = 62). However, time costs in a series of preparations such as numerous centrifugations, wash, ethanol/formic acid extraction were not included, making the approach infeasible in the clinical setting. A total of three antimicrobial agents were assessed for the *Streprococcus/Enterococci* species and the results showed an agreement of 89.7%, with 0% ofminor error, 8.7% of ME, and 16.7% of VME (*n* = 10) [12]. The average CA of 97.5% was also lower than when using our approach.

There are some unavoidable limitations in this study. About 4.3% of the samples were detected as polymicrobial, which further delayed ID and AST. This aspect was consistent with earlier investigations, suggesting that the incidence of polymicrobial bloodstream infection (BSI, the presence of at least two different pathogens in one set of blood cultures) varied from 6% to 32% of all BSI episodes [24]. Another limitation is that only a finite number of organisms were examined, although all selected organisms were commonly observed in general microbiology laboratories across the nation. A diverse set of isolates covering a range of species is recommended in the guidelines when verifying AST results. Lee et al. reported that the most common bacteremia pathogens during 2010–2015 in southern Taiwan were *Escherichia coli*, *Staphylococcus aureus, Streptococcus* species, *Klebsiella* species, *Enterococcus* species, *Pseudomonas* species, *Enterobacter* species, *Salmonella* species, *Acinetobacter* species, *and Proteus* species [2]. Our study included all of these microorganisms, covering the common bloodstream infection pathogens for bacterial ID and AST.

## 5. Conclusions

Taken together, the shortest incubation to identification time of the subculture method in our study showed a high percentage of category agreements (>90%). In addition, no extra extraction step is required compared to the other approaches. With further automation and process optimization, we propose a decrease in at least 12 h, which would allow for more timely ID and AST reports and ultimately benefit clinical outcomes for every infected individual. Further studies are needed to improve turnaround times and cost-effectiveness analyses of rapid ID and AST methods.

## Figures and Tables

**Table 1:** Gram-positive bacteria identified by the short-term incubation (4.5 h) were concordant to those from the conventional method.

	Short-Term Culture Followed by MALDI-TOF MS [*n* (%)]						Conventional
Organism	1.5 h	2 h	2.5 h	3 h	3.5 h	4.5 h	>24 h
*Staphylococcus* spp.	24 (54.4%)	29 (65.9%)	30 (68.2%)	37 (84.1%)	40 (90.9%)	44 (100%)	44
*Staphylococcus aureus*	18	19	19	22	22	22	22
*Staphylococcus capitis*	2	2	3	3	3	7	7
*Staphylococcus caprae*	1	2	0	2	2	2	2
*Staphylococcus epidermidis*	3	5	5	5	7	7	7
*Staphylococcus hominis*	0	1	2	2	2	2	2
*Staphylococcus warneri*	0	0	1	3	4	4	4
*Enterococcus* spp.	25 (86.2%)	27 (93.1%)	29 (100%)	29 (100%)	29 (100%)	29 (100%)	29
*Enterococcus faecalis*	13	13	14	14	14	14	14
*Enterococcus faecium*	11	13	14	14	14	14	14
*Enterococcus gallinarum*	1	1	1	1	1	1	1
*Streptococcus* spp.	20 (66.7%)	14 (46.7%)	24 (80.0%)	15 (50.0%)	30 (100%)	30 (100%)	30
*Streptococcus agalactiae*	9	9	9	9	9	9	9
*Streptococcus anginosus*	1	-	2	-	4	4	4
*Streptococcus constellatus*	2	-	0	-	4	4	4
*Streptococcus dysgalactiae*	3	-	7	-	7	7	7
*Streptococcus gallolyticus*	3	3	3	3	3	3	3
*Streptococcus oralis*	0	0	1	1	1	1	1
*Streptococcus salivarius*	1	1	1	1	1	1	1
*Streptococcus suis*	1	1	1	1	1	1	1
Total	69 (67.0%)	70 (68.0%)	83 (80.6%)	81 (78.6%)	99 (96.1%)	103 (100%)	103 (100%)

**Table 2:** Gram-negative bacteria identified by the short-term incubation (3.5 h) were concordant to those from the conventional method.

	Short-Term Incubation Followed by MALDI-TOF MS					Conventional
Organism	1.5 h	2 h	2.5 h	3 h	3.5 h	>24 h
Enterobacterales						
*Citrobacter* spp.	3 (75.0%)	3 (75.0%)	3 (75.0%)	4 (100%)	4 (100%)	4
*Citrobacterbraakii*	0	0	0	1	1	1
*Citrobacterkoseri*	3	3	3	3	3	3
*Enterobacter* spp.						
*Enterobacter cloacae*	3 (75.0%)	3 (75.0%)	4 (100%)	4 (100%)	4 (100%)	4
*Escherichia* spp.	17 (77.3%)	21 (95.5%)	22 (100%)	22 (100%)	22 (100%)	22
*Escherichia coli*	17	21	22	22	22	22
*Klebsiella* spp.	10 (83.3%)	11 (91.7%)	12 (100%)	12 (100%)	12 (100%)	12
*Klebsiellaaerogenes*	2	2	2	2	2	2
*Klebsiellaoxytoca*	3	3	4	4	4	4
*Klebsiella pneumoniae*	5	6	6	6	6	6
*Leclercia* spp.	3 (100%)	3 (100%)	3 (100%)	3 (100%)	3 (100%)	3
*Leclerciaadecarboxylata*	3	3	3	3	3	3
*Morganella* spp.	4 (100%)	4 (100%)	4 (100%)	4 (100%)	4 (100%)	4
*Morganellamorganii*	4	4	4	4	4	4
*Proteus* spp.	3 (100%)	3 (100%)	3 (100%)	3 (100%)	3 (100%)	3
*Proteus mirabilis*	3	3	3	3	3	3
*Salmonella* spp.	3 (50.0%)	4 (66.7%)	5 (83.3%)	6 (100%)	6 (100%)	6
*Salmonella* spp.	3	4	5	6	6	6
*Serratia* spp.	2 (66.7%)	2 (66.7%)	2 (66.7%)	1 (33.3%)	1 (33.3%)	3
*Serratiamarcescens*	2	2	2	1	1	2
*Serratiaureilytica*	0	0	0	0	0	1
Subtotal	48 (78.7%)	54 (88.5%)	58 (95.1%)	59 (96.7%)	59 (96.7%)	61
Non-Enterobacterales						
*Acinetobacter* spp.	6 (100%)	5 (83.3%)	5 (83.3%)	6 (100%)	6 (100%)	6
*Acinetobacter baumannii*	4	3	3	4	4	4
*Acinetobacter johnsonii*	2	2	2	2	2	2
*Aeromonas* spp.	2 (100%)	2 (100%)	2 (100%)	2 (100%)	2(100%)	2
*Aeromonascaviae*	1	1	1	1	1	1
*Aeromonashydrophila*	1	1	1	1	1	1
*Pseudomonas* spp.	4 (66.7%)	5 (83.3%)	4 (66.7%)	6 (100%)	6 (100%)	6
*Pseudomonas aeruginosa*	4	5	4	6	6	6
*Stenotrophomonas* spp.	2 (100%)	2 (100%)	2 (100%)	2 (100%)	2 (100%)	2
*Stenotrophomonasmaltophilia*	2	2	2	2	2	2
Subtotal	14(87.5%)	14 (87.5%)	13 (81.3%)	16 (100%)	16 (100%)	16
Total	62 (80.5%)	68 (88.3%)	71 (92.2%)	75 (97.4%)	75 (97.4%)	77

**Table 3:** Except for CoNS ^1^, high category agreements for the antimicrobial agent selection were identified in Gram-positive bacteria which were examined by the short-term (4.5 h) incubation with the BD Phoenix M50 AST system ^2^.

Antimicrobial Agent	Category Agreement				Very Major Error				Major Error				Minor Error				Total	
	All		All-CoNS		All		All-CoNS		All		All-CoNS		All		All-CoNS		All	All-CoNS
	*n*	%	*n*	%	*n*/Resistant	%	*n*/Resistant	%	*n*/Susceptible	%	*n*/Susceptible	%	*n*/Total	%	*n*/Total	%	*n*	%
Ampicillin	29	100	29	100	0/14	0	0/14	0	0/15	0	0/15	0	0/29	0	0/29	0	29	29
Clindamycin	38	86.4	21	95.5	2/13	15.4	0/4	0	3/31	9.7	0/18	0	1/44	2.3	1/22	4.5	44	22
Fusidic Acid	39	88.6	22	100	4/7	57.1	0/0	0	1/37	2.7	0/22	0	0/44	0	0/22	0	44	22
Gentamicin	33	75.0	20	90.9	2/19	10.5	0/9	0	5/23	21.7	1/12	8.3	4/44	9.1	1/22	4.5	44	22
Gentamicin-Synergy	28	96.6	28	96.6	0/8	0	0/8	0	1/21	4.8	1/21	4.8	0/29	0	0/29	0	29	29
Minocycline	42	95.5	22	100	1/1	100	0/0	0	0/41	0	0/21	0	1/44	2.3	0/22	0	44	22
Oxacillin	22	100	22	100	0/10	0	0/10	0	0/12	0	0/12	0	0/22	0	0/22	0	22	22
Penicillin G	63	98.4	48	98.0	0/48	0	0/33	0	1/16	6.3	1/16	6.3	0/64	0	0/49	0	64	49
Teicoplanin	69	95.8	49	96.1	0/9	0	0/9	0	2/63	2.3	1/42	2.4	1/72	1.4	1/51	2.0	72	51
Trimethoprim-Sulfamethoxazole	36	81.8	20	90.9	2/7	28.6	0/2	0	5/36	13.9	2/20	10.0	1/44	2.3	0/22	0	44	22
Vancomycin	28	96.6	28	96.6	0/10	0	0/10	0	1/19	5.3	1/19	5.3	0/29	0	0/29	0	29	29
Total	427	91.8	309	96.9	11/146	7.5	0/99	0	19/314	6.1	7/218	3.2	8/465	1.7	3/319	0.9	465	319

^1^ Coagulase-negative *Staphylococci*: CoNS; ^2^ Compared to the conventional method [overnight-incubation colonies on disk diffusion of antimicrobial susceptibility tests (AST)]; All-CoNS: all bacteria except for (minus) CoNS.

**Table 4:** High category agreements for the antimicrobial agent selection were identified in *Streptococcus* spp. isolates which were examined by the short-term (3.5 h) incubation with the BD Phoenix M50 AST system ^1^.

Antimicrobial Agent	Category Agreement		Very Major Error		Major Error		Minor Error		Total
	*n*	%	*n*/Resistant	%	*n*/Susceptible	%	*n*/total	%	*n*
Ampicillin	16	100	0/0	0	0/16	0	0/16	0	16
Ceftriaxone	29	96.7	0/0	0	1/30	3.3	0/30	0	30
Clindamycin	28	93.3	0/9	0	2/21	9.5	0/30	0	30
Penicillin G	16	100	0/0	0	0/16	0	0/16	0	16
Vancomycin	30	100	0/0	0	0/30	0	0/3	0	30
Total	119	97.5	0/9	0	3/113	2.7	0/122	0	122

^1^ Compared to the conventional method [overnight-incubation colonies on disk diffusion of antimicrobial susceptibility tests (AST)].

**Table 5:** High category agreements for the antimicrobial agent selection were identified in Gram-negative bacteria which were examined by the short-term (3.5 h) incubation with the BD Phoenix M50 AST system ^1^.

Antimicrobial Agent	Category Agreement		Very Major Error		Major Error		Minor Error		Total
	*n*	%	*n*/Resistant	%	*n*/Susceptible	%	*n*/Total	%	*n*
Amikacin	17	94.4	0/4	0	0/14	0	1/18	5.6	18
Ampicillin	54	88.5	1/45	2.2	3/14	21.4	3/61	4.9	61
Cefazolin	46	83.6	3/33	9.1	1/11	9.1	5/55	9.1	55
Ceftazidime	67	89.3	0/17	0	0/55	0	8/75	10.7	75
Ceftriaxone	63	100	0/14	0	0/49	0	0/63	0	63
Ciprofloxacin	26	100	0/16	0	0/9	0	0/26	0	26
Colistin	0	0	0/0	0	0/0	0	0/0	0	0
Ertapenem	54	94.7	0/0	0	1/54	1.9	2/57	3.5	57
Gentamicin	66	98.5	0/10	0	1/57	1.8	0/67	0	67
Levofloxacin	8	80.0	0/0	0	0/10	0	2/10	20.0	10
Meropenem	14	100	0/5	0	0/9	0	0/14	0	14
Minocycline	7	87.5	1/1	100	0/4	0	0/8	0	8
Piperacillin-Tazobactam	63	94.0	0/5	0	0/57	0	4/67	6.0	67
Tigecycline	7	70.0	0/0	0	0/5	0	3/10	30.0	10
Trimethoprim-Sulfamethoxazole	15	93.8	1/5	20.0	0/11	0	0/16	0	16
Total	507	92.7	6/155	3.9	6/359	1.7%	28/547	5.1	547

^1^ Compared to the conventional method [overnight-incubation colonies on disk diffusion of antimicrobial susceptibility tests (AST)].

**Table 6:** High category agreements for Gram–positive and –negative bacteria were identified in the short-term incubation of disk diffusion method and the BD Phoenix™ M50 AST compared to the conventional overnight incubation method.

Organisms	Category Agreement		Very Major Error		Major Error		Minor Error	
	*n*/Total	%	*n*/Resistant	%	*n*/Susceptible	%	*n*/total	%
Disk Diffusion Method ^1^								
Gram-positive								
Non-*Streptococcus*	328/342	95.9	3/104	2.9	8/236	3.4	3/342	0.9
*Streptococcus*	116/119	97.5	0/8	0	2/111	1.8	1/119	0.8
Gram negative	440/447	98.4	0/133	0	0/287	0	7/447	1.6
BD Phoenix™ M50 AST ^2^								
Gram-positive								
Non-*Streptococcus*	1357/1420	95.6	8/551	1.5	17/820	2.1	38/1420	2.7
*Streptococcus*	370/390	94.9	2/40	5.0	10/345	2.9	8/390	2.1
Gram-negative	1245/1286	96.8	3/355	0.8	13/876	1.5	25/1286	1.9

^1^ Compared to the conventional disk diffusion using overnight incubation samples. ^2^ Compared to the BD Phoenix™ M50 AST using overnight incubation samples (AST: antimicrobial susceptibility tests).

## Data Availability

The raw data used to support the conclusions of this article will be made available by the corresponding author without undue reservation to any qualified researcher.
